# Glucosamine Use and Its Association with Colorectal Cancer and Mortality: A Systematic Review and Meta-Analysis

**DOI:** 10.7150/jca.133131

**Published:** 2026-08-11

**Authors:** Yiling Yan, Manrong Xu, Ruixin Wang, Shengdi Lu, Yun Shen, Feng Liu

**Affiliations:** 1 Department of Gastroenterology, Shanghai Tenth People's Hospital, Tongji University School of Medicine, Shanghai, China.; 2 Department of Endocrinology, Shanghai Sixth People's Hospital, Shanghai Jiao Tong University, Shanghai, China.; 3 School of Public Health, Fudan University, Shanghai, China.; 4 Pennington Biomedical Research Center, Baton Rouge, LA, United States.; 5 Digestive Endoscopy Center, Shanghai Tenth People's Hospital, Tongji University School of Medicine, Shanghai, China.

**Keywords:** glucosamine, colorectal cancer, cardiovascular disease, mortality

## Abstract

**Background and Aim:**

This systematic review and meta-analysis aimed to evaluate whether habitual glucosamine use is associated with colorectal cancer risk and survival outcomes in the general population.

**Methods:**

Following PRISMA 2020 guidance, we systematically searched PubMed, Embase, Web of Science, and the Cochrane Database of Systematic Reviews from inception to January 10, 2026. Prospective cohort studies comparing glucosamine users with non-users and reporting adjusted effect estimates were eligible. Two reviewers independently extracted data and assessed study quality using the Newcastle-Ottawa Scale. Adjusted hazard ratios or relative risks were pooled using fixed- or random-effects models based on heterogeneity quantified with I².

**Results:**

From 347 records, 18 prospective cohort studies met eligibility criteria; five outcomes (colorectal cancer incidence, colorectal cancer-specific mortality, all-cause mortality, cardiovascular mortality, and overall cancer mortality) were quantitatively synthesized. Glucosamine use was associated with a lower incidence of colorectal cancer across 5 studies (pooled HR 0.87, 95% CI 0.82 to 0.92; I² = 36%). For colorectal cancer-specific mortality, the evidence was limited to only 2 studies with substantial heterogeneity (I² = 81%) and effect estimates in opposite directions, and the pooled estimate showed no clear association (pooled RR 0.99, 95% CI 0.57 to 1.71); this outcome should therefore be regarded as inconclusive. Glucosamine use was also associated with lower all-cause mortality in 4 studies (pooled HR 0.84, 95% CI 0.76 to 0.94; I² = 65%), lower cardiovascular mortality in 4 studies (pooled HR 0.82, 95% CI 0.75 to 0.89; I² = 44%), and a modest reduction in overall cancer mortality in 4 studies (pooled HR 0.94, 95% CI 0.91 to 0.98; I² = 7%). Results were stable in leave-one-out sensitivity analyses. All estimates represent associations derived from observational cohort studies and cannot establish causation.

**Conclusions:**

Current cohort evidence indicates that glucosamine use is associated with a modestly lower risk of developing colorectal cancer and with lower mortality from all causes, cardiovascular disease, and cancer overall. The evidence for colorectal cancer-specific mortality was based on only 2 studies with substantial heterogeneity and remains inconclusive. Because all included studies were observational, these findings represent associations rather than evidence of a causal protective effect. Given the modest effect sizes, healthy-user bias and residual confounding are plausible and possibly major explanations for the observed associations, and even a small degree of unmeasured confounding could account for part or all of them. Well-designed studies with improved exposure characterization, and ideally causal designs such as Mendelian randomization, are needed to determine whether these associations are causal and clinically meaningful.

## Background

Colorectal cancer is a major cause of cancer morbidity and mortality worldwide. In 2022 there were about 1.9 million new cases and roughly 0.9 million deaths 1. Prevention remains central because many tumors arise over long latent periods. At the same time, complementary health approaches and nonprescription supplements are widely used, particularly among adults at ages when colorectal cancer risk is increasing 2. In population surveys of older adults, glucosamine is among the most commonly reported specialty supplements, often taken daily and for years for osteoarthritis, creating sustained exposure in groups at highest colorectal cancer risk 3.

Several biological pathways connect glucosamine to colorectal carcinogenesis. Inflammation supports early neoplasia and tumor progression, and it is a plausible prevention target. Experimental work shows glucosamine can suppress inflammatory signaling, including inhibition of nuclear factor kappa B activation in cytokine-stimulated human osteoarthritic chondrocytes 4. Human evidence is directionally consistent. In a randomized, placebo-controlled crossover trial in healthy overweight adults, glucosamine plus chondroitin lowered circulating C-reactive protein and shifted plasma proteomic signatures toward reduced cytokine activity 5. In observational analyses, glucosamine and chondroitin use has also been associated with lower C-reactive protein concentrations, with variation by formulation and dose 6. These findings support a credible anti-inflammatory rationale for colorectal cancer risk reduction.

A second, more ambivalent mechanism centers on the hexosamine biosynthetic pathway. Glucosamine can increase intracellular UDP-N-acetylglucosamine and protein-O-GlcNAcylation, a nutrient-sensing modification that regulates transcription, signal transduction, and stress responses. In colorectal cancer, altered O-GlcNAcylation and related enzymes have been linked to proliferation, invasion, and therapy response, implying that supplementation could influence carcinogenesis or progression in a context-dependent manner 7. These countervailing pathways provide a biologic explanation for why population-level associations might be heterogeneous.

Epidemiologic evidence is therefore challenging to interpret. Prospective cohorts have reported inverse associations between glucosamine use and colorectal cancer incidence in VITAL, in the Nurses' Health Study and Health Professionals Follow-up Study, and in the Cancer Prevention Study II Nutrition Cohort 8-10. In contrast, analyses in UK Biobank found no clear overall association and suggested possible modification by screening history and early follow-up time 11. Mortality studies have reported lower all-cause and cause-specific mortality among regular users in large cohorts 12, 13. Yet unexpectedly strong protective estimates raise concern for residual confounding, healthy-user behavior, and selection processes. A dedicated bias analysis has argued that collider stratification and related selection bias could plausibly produce spurious benefit signals in observational glucosamine research 14. Recent reviews and meta-analyses have helped map the field, but they commonly combine glucosamine with chondroitin, focus on incidence rather than colorectal cancer mortality, or predate newer cohort updates 15, 16. Specifically, the systematic review by Khan et al. did not perform a quantitative synthesis, and the meta-analysis by Liu et al. pooled glucosamine together with chondroitin and did not separately address colorectal cancer-specific mortality 15, 16. Neither incorporated the more recent large-cohort updates, including the UK Biobank analyses and other prospective cohorts published from 2022 onward. A focused synthesis that isolates glucosamine from chondroitin, incorporates these newer cohorts, and jointly evaluates both colorectal cancer incidence and colorectal cancer-specific mortality within a single framework is therefore still needed and represents the principal contribution of the present review.

We conducted a systematic review and meta-analysis of prospective cohort studies to quantify the association between glucosamine use and colorectal cancer incidence and mortality outcomes, and to explore sources of heterogeneity in line with PRISMA principles.

Our scientific hypothesis is that habitual glucosamine use is associated with a lower risk of colorectal cancer and lower mortality, predominantly through systemic anti-inflammatory pathways, while any association with colorectal cancer-specific mortality may be smaller and more sensitive to screening patterns and residual confounding.

## Method

### Registration and Protocol

The study protocol was prospectively registered with the International Prospective Register of Systematic Reviews, PROSPERO (CRD420261303799). This study was conducted as a systematic review and meta-analysis, synthesizing evidence from published prospective cohort studies to evaluate the association between glucosamine use and multiple clinical outcomes in the general population. The methodology followed established recommendations for systematic reviews and meta-analyses, and the reporting adhered to the PRISMA 2020 guidelines 17.

### Information Sources and Search Strategy

A comprehensive literature search was conducted to evaluate the association between glucosamine supplementation and multiple clinical outcomes in the general population. Relevant studies were identified through systematic searches of PubMed, Embase, Web of Science, and the Cochrane Database of Systematic Reviews from inception to January 10, 2026. The search strategy combined Medical Subject Headings and free-text terms related to glucosamine use, prospective cohort studies, and clinical outcomes. Key search terms included "glucosamine," "glucosamine sulfate," "glucosamine hydrochloride," "prospective cohort," "incidence," "mortality," "cancer," and "cardiovascular disease." Boolean operators and database-specific syntax were applied to ensure comprehensive retrieval. The full search strategy is provided in Supplementary Table 1.

### Inclusion and Exclusion Criteria

Inclusion Criteria. Studies were considered eligible for inclusion if they met all of the following criteria: (1) assessed glucosamine use as the primary exposure; (2) included a comparison group of non-users; (3) reported adjusted effect estimates, including hazard ratios, relative risks, or incidence rate ratios, with 95% confidence intervals or sufficient data to derive them; and (4) were conducted in general population cohorts rather than disease-specific patient populations.

Exclusion Criteria. Cross-sectional studies, case-control studies, randomized trials, reviews, editorials, conference abstracts, animal or in vitro studies, and studies conducted exclusively in patient populations were excluded. Studies that did not report relevant clinical outcomes, lacked a clear definition of glucosamine exposure, or did not provide extractable effect estimates were also excluded. For multiple publications from the same cohort, we retained the most recent or most comprehensive report.

### Data Extraction and Handling

Data extraction was independently conducted by two reviewers using a standardized form. Extracted information included author and publication year, characteristics of included primary studies, population and study design, definition and measurement of glucosamine exposure and comparator, outcome measures, and adjusted effect estimates with corresponding 95% confidence intervals.

The primary outcomes of this review were colorectal cancer incidence and colorectal cancer-specific mortality. Secondary outcomes were all-cause mortality, cardiovascular mortality, and overall cancer mortality. A quantitative meta-analysis was performed only for these five outcomes, each of which was reported by at least two included cohorts using compatible effect measures. The remaining outcomes captured by the included cohorts, including other site-specific cancers, incident gout, type 2 diabetes, coronary heart disease, chronic obstructive pulmonary disease, sepsis, COVID-19 outcomes, and neurodegenerative outcomes, were reported by single cohorts or were not amenable to pooling; these were therefore summarized qualitatively and were not entered into any pooled analysis. When multiple estimates or follow-up periods were reported, the most fully adjusted model and the longest follow-up were preferentially extracted.

Effect estimates were harmonized as hazard ratios or relative risks. When necessary, standard errors and variances were derived from reported confidence intervals. To account for potential heterogeneity across studies, pooled analyses were subsequently performed using a random-effects model. Disagreements in data extraction were resolved through discussion or consultation with a third reviewer.

### Statistical Analysis

All statistical analyses were performed using Review Manager (RevMan, version 5.4). Adjusted effect estimates, including hazard ratios and relative risks, with corresponding 95% confidence intervals were extracted from eligible prospective cohort studies. For studies reporting relative risks or incidence rate ratios, these were treated as approximations of hazard ratios and pooled together. This approach is widely accepted in meta-analyses of cohort studies when absolute event rates are low, follow-up durations are broadly comparable across studies, and the outcome is relatively rare, conditions under which these measures converge closely. We acknowledge that this approximation may introduce a small degree of imprecision, particularly for more common outcomes, and this is noted as a limitation. When necessary, standard errors were derived from reported confidence intervals.

For each outcome, only the subset of cohorts that reported that specific outcome with an extractable adjusted effect estimate and 95% confidence interval contributed to the corresponding pooled analysis. Because the included cohorts were designed to address different research questions, they reported different endpoints; consequently, the number of studies contributing to each pooled analysis varied across outcomes. The studies contributing to each pooled analysis are listed in Supplementary Table 5. Heterogeneity among studies was assessed using Cochran's Q test and quantified using the I² statistic. A fixed-effect model was applied when heterogeneity was low (I² ≤ 50%), whereas a random-effects model using the DerSimonian-Laird method was used when heterogeneity was substantial (I² > 50%). Pooled effect estimates were calculated using the generic inverse variance method.

Where applicable, leave-one-out sensitivity analyses were performed to evaluate the robustness of the pooled estimates. Because all included studies received Newcastle-Ottawa Scale scores of 7 or higher, formal quality-based stratification was not pursued; however, study quality was considered qualitatively when interpreting pooled estimates. Statistical significance was defined as a two-sided p value < 0.05. Results were summarized in tabular form, and forest plots for each outcome were generated and presented in the Figures.

### Risk of Bias Assessment and Methodological Quality

The methodological quality of the included prospective cohort studies was assessed using the Newcastle-Ottawa Scale. This tool evaluates three domains: Selection (4 stars), Comparability (2 stars), and Outcome (3 stars), with a maximum score of 9 stars 18. Each study was independently assessed by two reviewers, and disagreements were resolved through discussion or consultation with a third reviewer. Studies scoring 7 or more stars were considered to be of high quality, 5 to 6 stars as moderate quality, and fewer than 5 stars as low quality. No studies were excluded based solely on Newcastle-Ottawa Scale score; however, study quality was considered when interpreting the pooled estimates.

## Results

### Literature Retrieval

**Figure 1:**
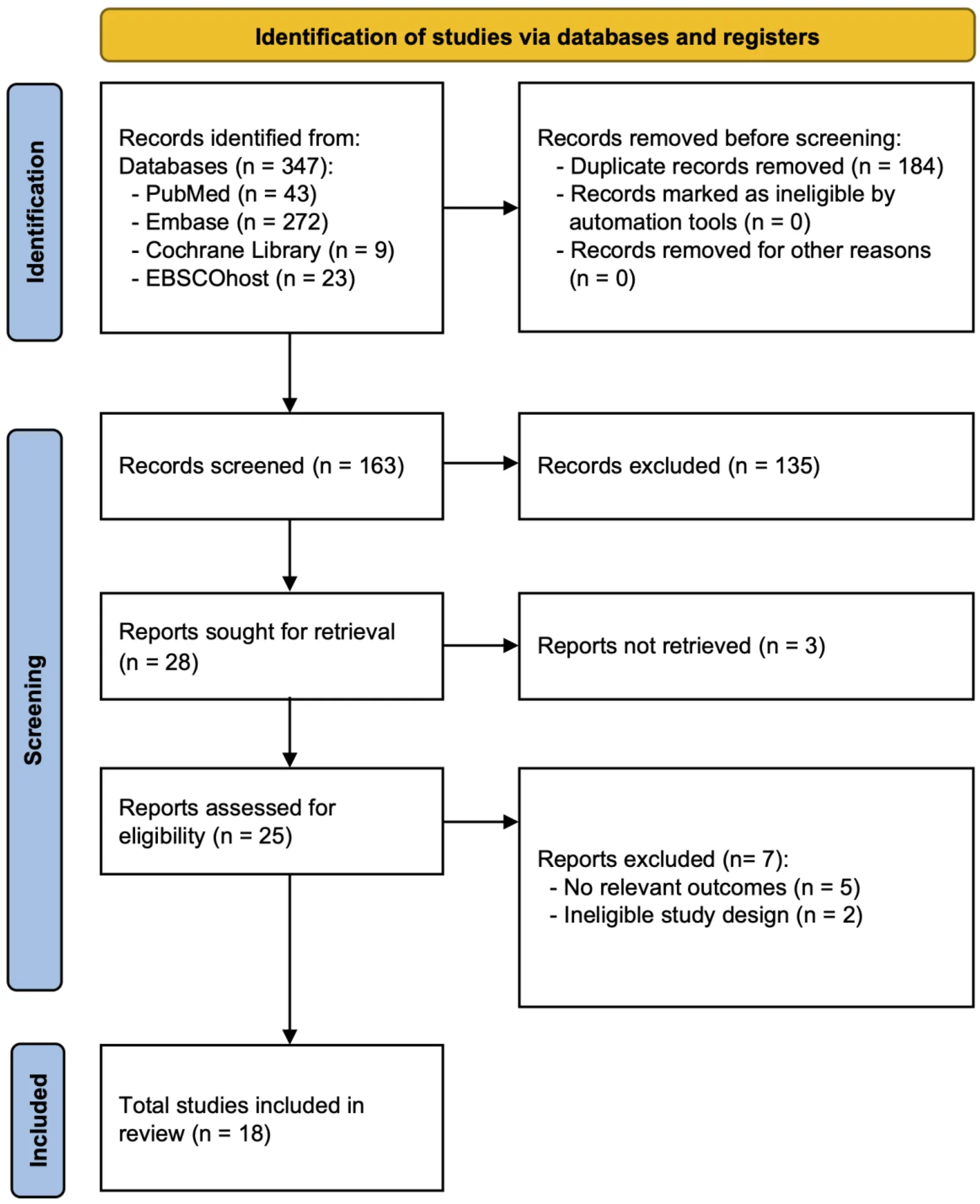
Flowchart of included studies.

The PRISMA 2020 flow diagram is presented in Figure 1. A total of 347 records were identified through a systematic literature search, and after stepwise screening, 18 prospective cohort studies were included 8-13, 19-30. These studies evaluated the association between glucosamine use and multiple clinical outcomes in the general population, including overall and site-specific cancers, cardiovascular outcomes, all-cause and cause-specific mortality, incident gout, and neurodegenerative outcomes. The detailed characteristics of the included studies are summarized in Table 1.

### Methodological Quality

Across Newcastle-Ottawa Scale domains, most studies received full stars for representativeness of the exposed cohort, selection of the non-exposed cohort, comparability, and outcome assessment. Partial deductions were mainly observed for exposure ascertainment 8, 9, 19, 21 and adequacy of follow-up 19, 21, reflecting reliance on self-reported exposure and incomplete reporting of follow-up losses in some earlier cohorts (Table 2).

### Meta-Analysis Results

**Figure 2:**
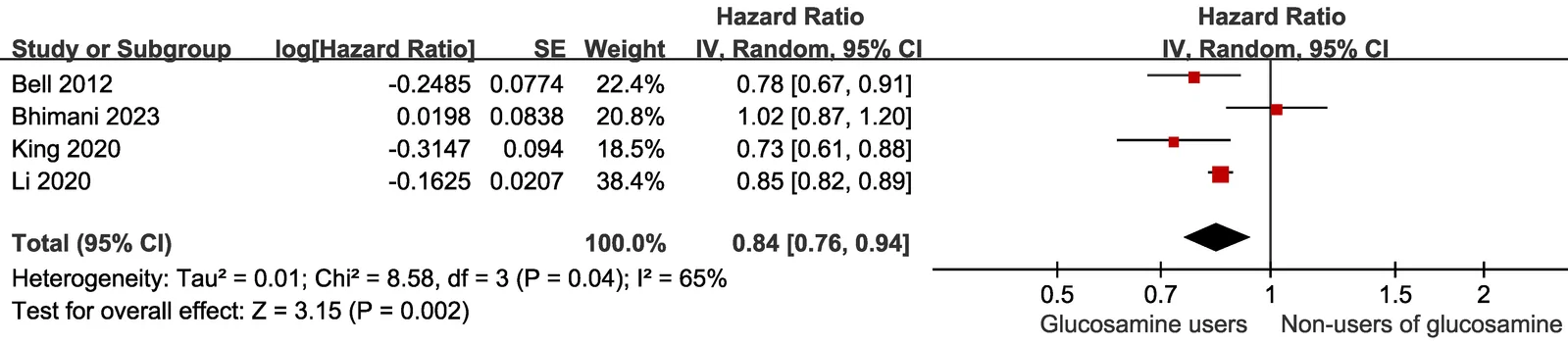
Forest plot of the association between glucosamine use and all-cause mortality.

All-Cause Mortality. Four prospective cohort studies contributed data to the analysis of all-cause mortality. Glucosamine use was associated with a lower risk of all-cause mortality (pooled HR = 0.84, 95% CI 0.76 to 0.94). Moderate heterogeneity was observed across studies (I² = 65%), and a random-effects model was applied. Leave-one-out sensitivity analysis excluding each study in turn did not materially alter the pooled estimate, suggesting robustness. Figure 2 shows the forest plot.

**Figure 3:**
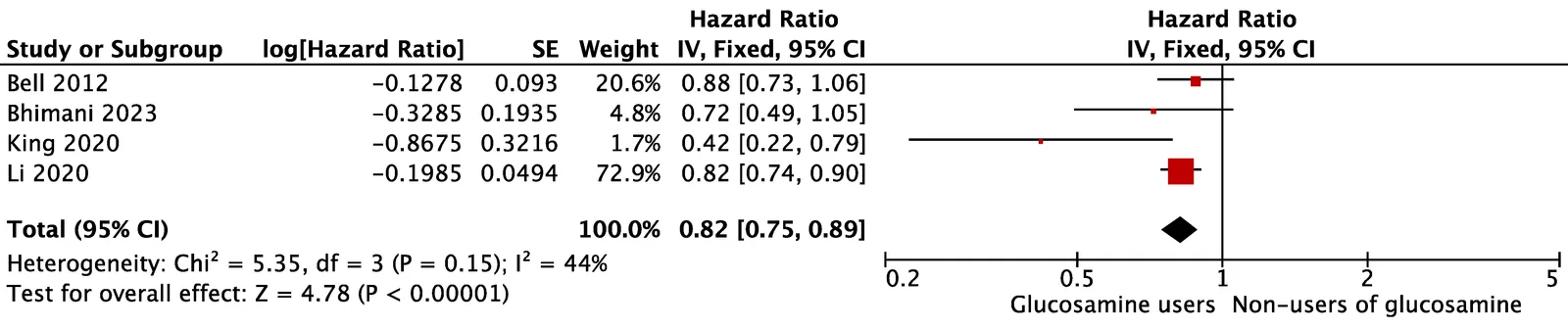
Forest plot of the association between glucosamine use and cardiovascular mortality.

Cardiovascular Mortality. Four prospective cohort studies reported cardiovascular mortality. Glucosamine use was associated with a lower risk of cardiovascular mortality (pooled HR = 0.82, 95% CI 0.75 to 0.89). Between-study heterogeneity was low (I² = 44%), and a fixed-effect model was applied. Leave-one-out sensitivity analyses indicated that no single study had a disproportionate influence on the pooled estimate. Figure 3 shows the forest plot.

**Figure 4:**
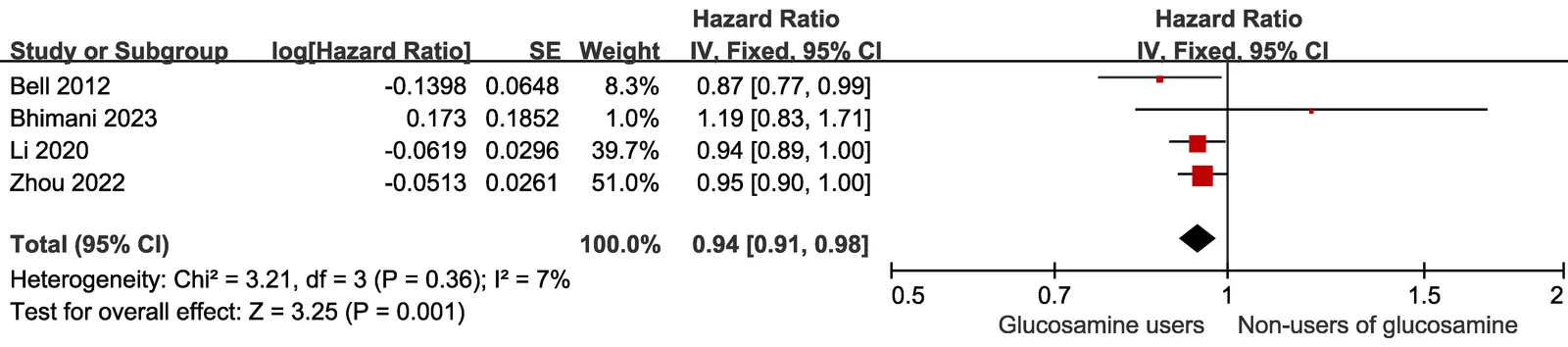
Forest plot of the association between glucosamine use and overall cancer mortality.

Overall, Cancer Mortality. Four studies were included in the meta-analysis of overall cancer mortality. Glucosamine use was associated with a modest but statistically significant reduction in cancer mortality (pooled HR = 0.94, 95% CI 0.91 to 0.98). Heterogeneity was low (I² = 7%), and a fixed-effect model was applied. The direction of the association was consistent across most cohorts. Figure 4 shows the forest plot.

**Figure 5:**
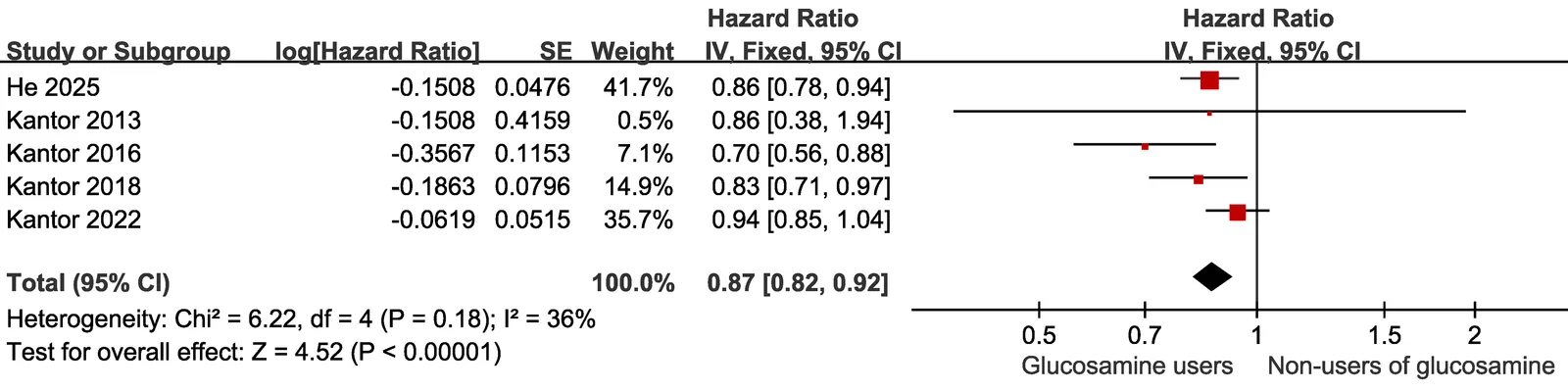
Forest plot of the association between glucosamine use and colorectal cancer incidence.

Colorectal Cancer Incidence. Five prospective cohort studies examined colorectal cancer incidence. Glucosamine use was associated with a lower incidence of colorectal cancer (pooled HR = 0.87, 95% CI 0.82 to 0.92). Heterogeneity was low (I² = 36%), and a fixed-effect model was applied. Figure 5 shows the forest plot.

**Figure 6:**
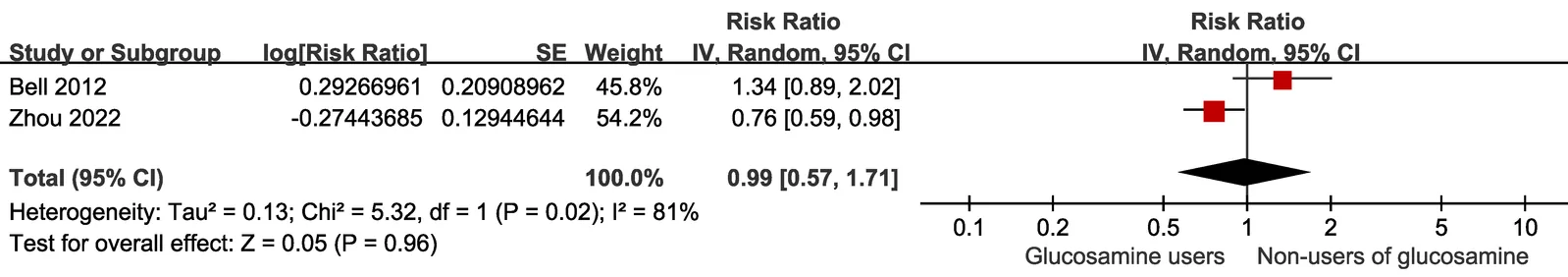
Forest plot of the association between glucosamine use and colorectal cancer mortality.

Colorectal Cancer Mortality. Only two cohort studies contributed data on colorectal cancer-specific mortality, and the evidence base for this outcome was therefore very limited. The two estimates were in opposite directions (Bell 2012, RR 1.34, 95% CI 0.89 to 2.02; Zhou 2022, RR 0.76, 95% CI 0.59-0.98), and between-study heterogeneity was substantial (I² = 81%). The pooled estimate showed no clear association (pooled RR = 0.99, 95% CI 0.57 to 1.71), and a random-effects model was applied. Given that this analysis rested on only two discordant studies with a wide confidence interval and high heterogeneity, the pooled estimate is statistically fragile and should be regarded as inconclusive rather than as evidence of no effect. Figure 6 shows the forest plot.

### Sensitivity Analyses

Leave-one-out sensitivity analyses were performed for all outcomes. The pooled estimates remained stable after sequentially excluding each study, indicating that no single study unduly influenced the overall results.

### Publication Bias

Formal assessment of publication bias using Egger's test or funnel plots was not performed because fewer than 10 studies were available for each pooled outcome, which limits the reliability of such tests. This constraint means that the possibility of selective reporting or non-publication of null findings cannot be excluded and should be considered when interpreting the pooled estimates.

## Discussion

This systematic review and meta-analysis evaluated the association between habitual glucosamine use and colorectal cancer incidence, colorectal cancer-specific mortality, and broader mortality outcomes. The pooled estimates consistently indicated that glucosamine use was associated with modestly lower risks for several endpoints; however, the magnitude of these associations was generally small, and the findings should be interpreted strictly as associations within the constraints of observational evidence rather than as evidence of a causal protective effect. As detailed below, healthy-user bias and residual confounding are likely to be major, rather than merely incidental, explanations for the observed associations.

To our knowledge, this is the first synthesis to isolate glucosamine from chondroitin and to evaluate colorectal cancer incidence and colorectal cancer-specific mortality jointly while also incorporating the recent large-cohort updates. For the five outcomes that were quantitatively synthesized, the pooled analyses suggested a consistent pattern of inverse associations. Overall cancer mortality showed a small but statistically significant association (pooled HR = 0.94, 95% CI 0.91 to 0.98) with minimal heterogeneity, indicating consistency across studies. All-cause mortality was also lower among glucosamine users, though moderate heterogeneity across studies warrants caution. Cardiovascular mortality showed a similar inverse association (pooled HR = 0.82, 95% CI 0.75 to 0.89), which may be consistent with the reported anti-inflammatory and metabolic properties of glucosamine, although causal inference is not possible from observational data alone. Colorectal cancer incidence was lower among glucosamine users (pooled HR = 0.87, 95% CI 0.82 to 0.92), but variability across studies likely reflects differences in exposure definitions, follow-up duration, and covariate adjustment.

Beyond the pooled estimates, the pattern of associations can be interpreted in light of the underlying biology, and the two mechanistic frameworks outlined earlier help to contextualize rather than merely quantify the findings. The most consistent inverse associations were observed for endpoints in which chronic inflammation is an established driver, namely colorectal cancer incidence and overall cancer and cardiovascular mortality. This is coherent with experimental evidence that glucosamine suppresses nuclear factor kappa B (NF-κB) signaling in a dose-dependent manner by inhibiting degradation of its inhibitory subunit IκB, thereby preventing nuclear translocation of the p50 and p65 subunits and reducing downstream cyclooxygenase-2 expression and prostaglandin E2 synthesis 4, and with animal data showing attenuation of experimental colitis 8. Human data are directionally supportive but modest: a randomized placebo-controlled trial and observational biomarker studies have linked glucosamine use to lower C-reactive protein and related inflammatory markers, though effects were small and not uniformly significant 5,6. Importantly, this anti-inflammatory framework does not operate in isolation. The countervailing hexosamine biosynthetic pathway, through which glucosamine augments protein O-GlcNAcylation and may in some contexts favor tumor proliferation and progression 7, offers a theoretical explanation for why a uniform protective effect should not be expected and why the incidence signal was not mirrored by a colorectal cancer-specific mortality benefit. Interpreted together, the biological and epidemiological evidence converges on a cautious inference: the data are compatible with a weak anti-inflammatory chemopreventive signal at the stage of tumor initiation, but the absence of a mortality gradient, the small effect sizes, and the opposing biology of the hexosamine pathway argue against a strong or uniform causal effect. Establishing causality would require evidence that current observational designs cannot provide, such as dose-response and duration-response relationships, mechanistic biomarker mediation analyses linking glucosamine exposure to reductions in NF-κB-driven inflammation and subsequent tumor outcomes, and, ideally, Mendelian randomization or adequately powered randomized trials with clinical endpoints.

While several of these pooled estimates reached conventional statistical significance, the absolute magnitude of the associations was small. Hazard ratios close to unity translate to modest absolute risk differences at the population level, which may have limited clinical impact for individual decision-making. Effect sizes in this range are particularly susceptible to residual confounding. Even a small degree of unmeasured confounding or systematic bias could shift these estimates toward or across the null, potentially accounting for part or all of the observed associations. The distinction between statistical significance and clinical relevance is therefore critical. These findings should not be used to inform clinical recommendations in the absence of corroborating evidence from causal study designs.

A notable finding is the discrepancy between colorectal cancer incidence and colorectal cancer-specific mortality. While a statistically significant inverse association was observed for incidence, no such association was found for mortality. Several explanations merit consideration. First, glucosamine users, who tend to be more health-conscious, may be more likely to undergo routine colorectal screening. This could lead to earlier and more frequent detection of adenomas and early-stage tumors, thereby lowering observed incidence in adjusted models, but screen-detected tumors often carry a favorable prognosis regardless of supplement use. Such detection bias would produce an apparent incidence reduction without a corresponding mortality benefit. Second, the two studies reporting colorectal cancer mortality used different analytic strategies, adjustment sets, and population characteristics. These methodological differences limit direct comparability and likely contributed to the high between-study heterogeneity (I² = 81%). Third, with only two contributing studies, the evidence base was inherently fragile. The wide confidence interval and inconsistent direction of effect between studies preclude meaningful conclusions about this endpoint. This pattern weakens the biological plausibility of a direct protective effect of glucosamine on colorectal cancer-specific outcomes.

The broadly inverse associations observed across multiple and biologically diverse endpoints also raise concern for healthy-user bias. Individuals who voluntarily take dietary supplements such as glucosamine tend to differ systematically from non-users in ways that independently predict better health outcomes. They are more likely to be physically active, to have higher socioeconomic status, to maintain better dietary quality, and to engage with preventive healthcare services including cancer screening. Such confounding can persist even after multivariable adjustment or propensity score matching, because key behavioral and health-seeking variables are often measured imprecisely or omitted from analytic models. The pattern of protective associations across such diverse outcomes as cardiovascular mortality, respiratory disease, sepsis, and cancer is itself a recognized hallmark of healthy-user confounding and should not be attributed uncritically to a pharmacologic effect of glucosamine.

Among outcomes that were not meta-analyzed, habitual glucosamine use was associated with lower risk of chronic obstructive pulmonary disease, reduced sepsis incidence and sepsis-related mortality, and lower risk of COVID-19 hospitalization and mortality in individual UK Biobank studies 24, 26, 28. Metabolic and rheumatologic analyses found inverse associations with incident gout, type 2 diabetes, and coronary heart disease 22, 25. Neurological studies reported associations with reduced dementia risk 27. These findings, while broadly consistent with the direction observed for colorectal cancer outcomes, should be interpreted as hypothesis-generating rather than confirmatory.

Between-study heterogeneity may be partly explained by several methodological and population-level differences. Exposure definitions varied across studies, ranging from habitual use to regular use, with different recall periods and ascertainment methods. Screening behavior and healthcare access differed across cohorts, particularly between UK Biobank, VITAL, and NHANES-based studies. Covariate adjustment strategies were not uniform. Some studies adjusted for a comprehensive set of lifestyle and dietary confounders, while others used more limited models. Follow-up duration and population demographics also varied, each of which can influence point estimates. These factors likely contribute to the observed variability and should temper the certainty of any summary conclusions.

Several limitations should temper interpretation. First, all included studies were observational, so causal inference is not possible. Residual confounding remains a central concern, even in fully adjusted models. Diet quality, physical activity, adiposity, medication use, access to care, and colorectal screening history are not measured with the same precision across cohorts and may not be fully controlled. Healthy-user bias is also plausible, as individuals who choose long-term supplement use often engage in broader health-promoting behaviors and preventive care, which could partly explain lower observed incidence or mortality even after multivariable adjustment. Second, glucosamine exposure was self-reported in all included studies, typically assessed at a single baseline time point, with no information on changes in use, adherence, dose adjustments, or discontinuation during follow-up. This introduces nondifferential misclassification that would generally bias associations toward the null, but differential reporting among health-conscious individuals cannot be excluded. The inability to capture time-varying exposure limits the precision of exposure characterization and may attenuate observed associations, though bias in either direction cannot be fully ruled out. Third, glucosamine products are heterogeneous. Cohorts rarely distinguished glucosamine sulfate from glucosamine hydrochloride, and many participants used mixed formulations. Dose, frequency, and cumulative exposure were not standardized, limiting dose-response evaluation. Fourth, the evidence base for colorectal cancer-specific mortality was small, with only two studies contributing data, substantial between-study heterogeneity, and wide confidence intervals. This constrained meaningful subgroup analyses and limited formal assessment of publication bias. Fifth, formal publication bias assessment was not feasible for any outcome because fewer than 10 studies contributed to each pooled analysis, and the possibility of selective reporting or non-publication of null findings cannot be excluded.

## Conclusion

In pooled analyses of prospective cohorts, glucosamine use was associated with a modest reduction in colorectal cancer incidence and with small reductions in all-cause and overall cancer mortality. Evidence for colorectal cancer-specific mortality was based on only two studies with substantial heterogeneity and discordant results; it was heterogeneous, imprecise, and inconclusive, and it did not support a clear association. The observed effect sizes were small, so any absolute risk reduction is likely modest, and associations of this magnitude are particularly susceptible to healthy-user bias and residual confounding, which could plausibly account for part or all of the observed associations. These findings are compatible with an anti-inflammatory explanation, but they represent associations only, should not be interpreted as evidence of a causal protective effect, and are not practice-changing. Because the data are observational, residual confounding and healthy-user differences cannot be excluded, even in well-adjusted models. Glucosamine should not be recommended as a primary preventive agent for colorectal cancer at this time. If glucosamine is used for other indications, the current evidence does not suggest increased colorectal cancer risk, but it also does not justify initiation solely for cancer prevention. Future work should prioritize causal designs, including Mendelian randomization and randomized or pragmatic trial-based evaluations, with clear definition of formulation, dose, duration, adherence, and screening history to determine whether the observed associations are causal and clinically meaningful.

## Supplementary Material

Supplementary tables.

## Author contribution

Conceptualization: Feng Liu, Yun Shen & Shengdi Lu

Methodology: Shengdi Lu

Formal analysis: Ruixin Wang, Yiling Yan

Investigation: Yiling Yan

Data curation: Manrong Xu,

Writing - Original Draft: All authors

Writing - Review & Editing: Feng Liu, Yun Shen & Shengdi Lu

Visualization: Yiling Yan

Supervision: Feng Liu

## Figures and Tables

**Table 1:** Characteristics of Included studies.

Author (Surname, Year)	Population	Glucosamine non-users(n)	Glucosamine users (n)	Outcomes
Zhou 2022	UK Biobank participants aged 38-73	365421	88224	Connective/soft tissue cancer mortality; Esophageal cancer mortality; Head and neck cancer mortality; Kidney cancer mortality; Lung cancer mortality; Lymphoma/hematopoietic cancer mortality; Malignant melanoma mortality; Ovarian cancer mortality; Overall cancer mortality; Prostate cancer mortality; Rectal cancer mortality
Liu 2022	UK Biobank participants aged 37-73	6156	1247	Females using diuretics; Females not using diuretics; Incident gout (females); Incident gout (males); Incident gout (total population)
Meng 2023	UK Biobank participants aged 37-73	163031	42673	COVID-19 hospital admission; COVID-19 mortality; SARS-CoV-2 infection
Li 2020	UK Biobank general participants	16665	3217	All-cause mortality; CVD mortality; Cancer mortality; Digestive disease mortality; Respiratory disease mortality
Kantor 2022	UK Biobank participants aged 38-73	355,171	84,825	CRC risk (overall); CRC risk stratified by age; CRC risk stratified by sex
Bhimani 2023	NHANES 1999-2014 participants aged ≥20	4696	204	All-cause mortality; Cancer mortality; Cardiovascular mortality; Other-cause mortality
Kantor 2018	CPS-II Nutrition Cohort participants	1148	195	CRC incidence
Zhang 2022	UK Biobank participants mean age 56.5	391110	92593	COPD
King 2020	NHANES 1999-2010 US participants aged ≥40	16028	658	CVD mortality
Xu 2025	UK Biobank general participants aged 37-73	11,077	2,381	28-day mortality following sepsis; 60-day mortality following sepsis; 7-day mortality following sepsis; 90-day mortality following sepsis; Sepsis incidence
Li 2023	UK Biobank participants	49,596	13,834	Overall cancer incidence
He 2025	UK Biobank general participants	52,525	52,525	Overall cancer incidence
Li 2022	UK Biobank general participants aged 40-69	356,790	82,603	Lung cancer mortality (overall); Lung cancer incidence
Xu 2022	UK Biobank general participants age 37-73	5,418	1,413	Alzheimer's disease (AD); Incident dementia (overall); Joint effect: dementia (Model 3); Vascular dementia (VD)
Bell 2012	VITAL cohort, Washington State participants aged 50-76	61,769	12,937	All-cause mortality - current glucosamine use (any formulation); All-cause mortality - glucosamine without chondroitin; CVD mortality - current glucosamine use; Cancer mortality - current glucosamine use; ischemic heart disease mortality - current glucosamine use
Brasky 2011	VITAL cohort, community-dwelling participants in western Washington aged 50-76	691	116	Lung cancer
Kantor 2013	VITAL prospective cohort, western Washington participants aged 50-76	59,024	5,395	CRC incidence
Kantor 2016	NHS women + HPFS men	80,115	16,285	CRC incidence

CVD: Cardiovascular Disease; COPD: Chronic Obstructive Pulmonary Disease; CRC: Colorectal Cancer

**Table 2:** NOS quality assessment of included studies.

Study (Author, Year)	Selection (4)	Comparability (2)	Outcome (3)	Total (9)
Zhou 2022	4	2	3	9
Liu 2022	4	2	2	8
Meng 2023	4	2	2	8
Li 2020	4	2	3	9
Kantor 2022	4	2	3	9
Bhimani 2023	4	2	3	9
Kantor 2018	4	2	3	9
Zhang 2022	4	2	3	9
King 2020	4	2	3	9
Xu 2025	4	2	2	8
Li 2023	4	2	3	9
He 2025	4	2	2	8
Li 2022	4	2	3	9
Xu 2022	4	2	3	9
Bell 2012	3	2	2	7
Brasky 2011	3	2	2	7
Kantor 2013	4	2	2	8
Kantor 2016	4	2	2	8
