# Supplementary material for: Glucosamine Use and Its Association with Colorectal Cancer and Mortality: A Systematic Review and Meta-Analysis
Source: J Cancer. 2026 Aug 11;17(8):1520–30. doi: 10.7150/jca.133131 (PMC13505378; doi:10.7150/jca.133131)
Supplement: Supplementary file 1 [file jcav17p1520s1.pdf]

Supplementary Table 1 Search Strategies

Pubmed (Searching date: Jan 10, 2026)

#1 general population	“general population” [tiab] OR “population-based”[tiab] OR “community-based”[tiab]	420843
#2 Glucosamine	"Glucosamine"[Mesh] OR “Glucosamine”[tiab] OR “2-Amino-2-Deoxyglucose”[tiab] OR “2 Amino 2 Deoxyglucose”[tiab] OR “Glucosamine Sulfate”[tiab] OR “Dona”[tiab] OR “Dona S”[tiab] OR “Hespercorbin”[tiab] OR “Xicil”[tiab] OR “SYSADOA”[tiab] OR “Symptomatic slow-acting drugs in osteoarthritis”[tiab] OR “glucosamine sulphate”[tiab] OR “glucosamine hydrochloride”[tiab]	25940
#3	#1 AND #2	43

Embase

#1 general population	'general population'/exp<br>OR 'population based study'/exp<br>OR 'community based study'/exp<br>OR (general NEXT/1 population):ti,ab<br>OR 'population-based':ti,ab<br>OR 'community-based':ti,ab	1,218,341
#2 Glucosamine	'glucosamine'/exp<br>OR 'glucosamine sulfate'/exp<br>OR 'glucosamine hydrochloride'/exp<br>OR glucosamine:ti,ab<br>OR '2-amino-2-deoxyglucose':ti,ab<br>OR '2 amino 2 deoxyglucose':ti,ab	33962

	OR 'glucosamine sulfate':ti,ab<br>OR 'glucosamine sulphate':ti,ab<br>OR 'glucosamine hydrochloride':ti,ab<br>OR dona:ti,ab<br>OR 'dona s':ti,ab<br>OR hespercorbin:ti,ab<br>OR xicil:ti,ab<br>OR sysadoa:ti,ab<br>OR 'symptomatic slow-acting drugs in osteoarthritis':ti,ab	
#3	#1 AND #2	272

Cochrane Library

#1 general population	(general NEAR/1 population):ti,ab,kw<br>OR population-based:ti,ab,kw<br>OR community-based:ti,ab,kw	27753
#2 Glucosamine	[MeSH descriptor: Glucosamine explode all trees]<br>OR glucosamine:ti,ab,kw<br>OR "2-amino-2-deoxyglucose":ti,ab,kw<br>OR "2 amino 2 deoxyglucose":ti,ab,kw<br>OR "glucosamine sulfate":ti,ab,kw<br>OR "glucosamine sulphate":ti,ab,kw<br>OR "glucosamine hydrochloride":ti,ab,kw<br>OR dona:ti,ab,kw<br>OR "dona s":ti,ab,kw<br>OR hespercorbin:ti,ab,kw<br>OR xicil:ti,ab,kw	997

	OR sysadoa:ti,ab,kw<br>OR "symptomatic slow-acting drugs in osteoarthritis":ti,ab,kw	
#3	#1 AND #2	9

EBSCOhost

#1 general population	TIAB(general N1 population) OR TIAB(population-based) OR TIAB(community-based)	1,080,934
#2 Glucosamine	MH Glucosamine OR TIAB(glucosamine) OR TIAB("2-amino-2-deoxyglucose") OR TIAB("2 amino 2 deoxyglucose") OR TIAB("glucosamine sulfate") OR TIAB("glucosamine sulphate") OR TIAB("glucosamine hydrochloride") OR TIAB(dona) OR TIAB("dona s") OR TIAB(hespercorbin) OR TIAB(xicil) OR TIAB(sysadoa) OR TIAB("symptomatic slow-acting drugs in osteoarthritis")	16007
#3	#1 AND #2	23

Supplementary Table 2 PICO statement of all included studies

Title	Author (Surname + Year)	Country	Population	Baseline period	Follow-up (end date)	Follow-up duration	Glucosamine non-users(n)	Glucosamine users (n)	All outcomes
Association between glucosamine use and cancer mortality: A large prospective cohort study	Zhou 2022	UK	UK Biobank participants aged 38–73, cancer-free at baseline	2006–2010	1-Mar-21	Median 12.1 years	365421	88224	Connective/soft tissue cancer mortality； Esophageal cancer mortality； Head and neck cancer mortality； Kidney cancer mortality； Lung cancer mortality； Lymphoma/hematopoietic cancer mortality； Malignant melanoma mortality； Ovarian cancer mortality； Overall cancer mortality； Prostate cancer mortality； Rectal cancer mortality
Associations of habitual glucosamine supplementation	Liu 2022	UK	UK Biobank adults aged 37–73, free of gout at baseline	2006–2010	NR (as reported)	Median 12.1 years	6156	1247	Females using diuretics； Females not using diuretics； Incident gout (females)； Incident gout (males)；

with incident gout: a large population-based cohort study			(general population)						Incident gout (total population)
Associations of habitual glucosamine use with SARS-CoV-2 infection and hospital admission and death with COVID-19: Evidence from a large population based cohort study	Meng 2023	UK	UK Biobank adults aged 37–73 reinvited for SARS-CoV-2 antibody testing (general population; not a patient-only cohort)	2006–2010	30-Jan-20	Median 1.67 years	163031	42673	COVID-19 hospital admission； COVID-19 mortality； SARS-CoV-2 infection
Associations of regular glucosamine use with all-cause and cause-specific mortality: a large prospective cohort study	Li 2020	UK	UK Biobank general population; baseline 2006–2010	Recruitment 2006–2010	NR	Median 8.9 years	16665	3217	All-cause mortality； CVD mortality； Cancer mortality； Digestive disease mortality； Respiratory disease mortality
Glucosamine Use and Risk of Colorectal Cancer: Results from UK Biobank	Kantor 2022	UK	UK Biobank adults aged 38–73 at baseline (2006–2010); excluded prior	Baseline to end-of-study	31-Oct-15	Median 6.7 years (max 8.9)	355,171	84,825	CRC risk (overall)； CRC risk stratified by age；

			invasive CRC, missing glucosamine or key covariates						
Glucosamine and Chondroitin Use and Mortality Among Adults in the United States from 1999 to 2014	Bhimani 2023	USA	NHANES 1999–2014 adults aged ⩾ 20 (US civilian noninstitutionalized)	1999–2014	End of 2015 (NDI)	Median 8.1 years	4696	204	CRC risk stratified by sex
Glucosamine use and risk of colorectal cancer: results from the Cancer Prevention Study II Nutrition Cohort	Kantor 2018	USA	CPS-II Nutrition Cohort men and women; baseline 2001 questionnaire	Baseline 2001	30-Jun-11	Mean 8.3 years	1148	195	All-cause mortality; Cancer mortality； Cardiovascular mortality；
Glucosamine use, smoking and risk of incident chronic obstructive pulmonary disease: A large prospective cohort study	Zhang 2022	UK	UK Biobank community-dwelling adults (mean age 56.5y) without COPD at baseline	2006–2010	NR	Median 8.96 years	391110	92593	Other-cause mortality
Glucosamine/Chondroitin and Mortality	King 2020	USA	NHANES 1999–2010 US	1999–2010	31 Dec 2015 (Linked	Median 107 months	16028	658	CRC incidence

in a US NHANES Cohort			adults aged ⩾ 40		Mortality File)	(~8.9 years)			
Habitual Glucosamine Use and Risk of Sepsis: A 16-Year Follow-Up Study	Xu 2025	UK	UK Biobank general population (age 37–73 at recruitment 2006–2010)	2006–2010	31-Oct-22	Median 13.6 years	11,077	2,381	COPD
Regular Glucosamine Use May Have Different Roles in the Risk of Site-Specific Cancers: Findings from a Large Prospective Cohort	Li 2023	UK	UK Biobank participants free of cancer at baseline with complete glucosamine info	2006–2010	10 Nov 2021 (or event)	Median ~12.5 years	49,596	13,834	CVD mortality
Regular glucosamine supplementation and risk of age-related chronic diseases: evidence from a propensity score-matched cohort study	He 2025	UK	UK Biobank general population; included participants free of any NCDs at baseline	NR	31 Oct 2022 (HES)	Median 13.78 years	52,525	52,525	28-day mortality following sepsis； 60-day mortality following sepsis； 7-day mortality following sepsis； 90-day mortality following sepsis； Sepsis incidence

Relationship between glucosamine use and the risk of lung cancer: data from a nationwide prospective cohort study	Li 2022	UK	UK Biobank general population aged 40–69 (recruitment 2006–2010)	NR	21-May-20	Mean ~11.1 years	356,790	82,603	Overall cancer
The role of type 2 diabetes in the association between habitual glucosamine use and dementia: a prospective cohort study	Xu 2022	UK	UK Biobank general population (age 37–73) recruited 2006–2010	NR	30 Sept 2020 (or event)	Median 11 years	5,418	1,413	Overall cancer
Use of Glucosamine and Chondroitin in Relation to Mortality	Bell 2012	USA	VITAL cohort, Washington State community residents aged 50–76 (baseline 2000–2002)	NR	End of 2008 (mortality)	Mean 6.8 years; 526,403 person-years	61,769	12,937	Lung cancer mortality (overall)； Lung cancer incidence
Use of glucosamine and chondroitin and	Brasky 2011	USA	VITAL cohort, community-dwelling adults	NR	31-Dec-07	Mean ~6 years	691	116	Alzheimer’s disease (AD)；Incident dementia (overall)；Joint effect: dementia (Model

lung cancer risk in the VITAL cohort			aged 50–76 in western Washington						3)； Vascular dementia (VD)
Use of glucosamine and chondroitin supplements and risk of colorectal cancer	Kantor 2013	USA	VITAL prospective cohort, western Washington residents aged 50–76	NR	31-Dec-08	Mean 6.7 years	59,024	5,395	All-cause mortality – current glucosamine use (any formulation)； All-cause mortality – glucosamine without chondroitin； CVD mortality – current glucosamine use； Cancer mortality – current glucosamine use； Ischemic heart disease mortality – current glucosamine use
Use of glucosamine and chondroitin supplements in relation to risk of colorectal cancer: Results from the Nurses' Health Study and Health Professionals Follow-up Study	Kantor 2016	USA	Prospective cohorts (NHS women + HPFS men); baseline 2002	NR	NHS: 31 May 2010; HPFS: 31 Jan 2010	NR (cohort-specific)	80,115	16,285	Lung cancer

Supplementary Table 3 Summary of Data extraction

No	References	Author (Surname + Year)	Population	Intervention	Time span	Outcome type 1	Outcome	Number of control group	Number of intervention group	Outcome type 2	Outcome	Number of control group	Number of intervention group	Outcome type 3	Outcome	Number of control group	Number of intervention group	Outcome type 4	Outcome	Number of control group	Number of intervention group	Outcome type 5	Outcome	Number of control group	Number of intervention group
1	Association between glucosamine use and cancer mortality: A large prospective cohort study	Zhou 2022	UK Biobank participants aged 38–73, cancer-free at baseline	Habitual glucosamine use	2006–2010 baseline; follow-up to 1 Mar 2021 (median 12.1 years)	Overall cancer mortality	HR=0.95 (0.90–1.00); p=0.05	365421	88224	Bladder cancer mortality	IRR=0.86 (0.62–1.20); p=0.38	224	45	Brain cancer mortality	IRR=1.09 (0.89–1.34); p=0.40	485	127	Breast cancer mortality	IRR=1.03 (0.82–1.29); p=0.79	365	110	Colon cancer mortality	IRR=0.90 (0.74–1.11); p=0.33	525	127

	Association between glucosamine use and cancer mortality: A large prospective cohort study	Zhou 2022	UK Biobank participants aged 38–73, cancer-free at baseline	Habitual glucosamine use	2006–2010 baseline; follow-up to 1 Mar 2021 (median 12.1 years)	Connective/soft tissue cancer mortality	IRR=1.59 (1.00–2.52); p=0.05	66	28	Esophagus cancer mortality	IRR=0.94 (0.75–1.17); p=0.58	495	98	Head & neck cancer mortality	IRR=0.70 (0.41–1.20); p=0.19	116	16	Kidney cancer mortality	IRR=0.68 (0.49–0.95); p=0.02	255	43	Lung cancer mortality	IRR=0.84 (0.74–0.95); p=<0.01	1892	339
	Association between glucosamine use and cancer mortality: A large prospective cohort study	Zhou 2022	UK Biobank participants aged 38–73, cancer-free at baseline	Habitual glucosamine use	2006–2010 baseline; follow-up to 1 Mar 2021 (medi	Lymphoma/hematopoietic cancer mortality	IRR=1.13 (0.96–1.32); p=0.15	718	211	Malignant melanoma mortality	IRR=0.84 (0.55–1.28); p=0.41	135	27	Ovary cancer mortality	IRR=1.05 (0.83–1.32); p=0.67	317	105	Prostate cancer mortality	IRR=0.82 (0.65–1.05); p=0.11	455	85	Rectum cancer mortality	IRR=0.76 (0.59–0.98); p=0.04	410	76

					an 12.1 years)																				
	Association between glucosamine use and cancer mortality: A large prospective cohort study	Zhou 2022	UK Biobank participants aged 38–73, cancer-free at baseline	Habitual glucosamine use	2006–2010 baseline; follow-up to 1 Mar 2021 (median 12.1 years)	Skin cancer mortality	IRR=0.97 (0.32–2.93); p=0.96	20	4	Stomach cancer mortality	IRR=0.96 (0.72–1.29); p=0.81	271	61	Thyroid cancer mortality	IRR=0.79 (0.26–2.46); p=0.69	16	4	Uterus cancer mortality	IRR=0.84 (0.56–1.27); p=0.41	109	31				
2	Associations of habitual glucosamine supplementation with incident gout: a large	Liu 2022	UK Biobank adults aged 37–73, free of gout at baseline	Habitual glucosamine use	Baseline 2006–2010; median	Incident gout (total population)	HR=0.97 (0.91–1.04)	6156	1247	Incident gout (females)	HR=0.81 (0.71–0.92)	1375	343	Incident gout (males)	HR=1.05 (0.97–1.13)	4781	904	Females + diuretics	HR=0.64 (0.50–0.81)	908	206	Females without diuretics	HR=0.89 (0.77–1.03)	467	83

	population-based cohort study		(general population)		follow-up 12.1 years																				
3	Associations of habitual glucosamine use with SARS-CoV-2 infection and hospital admission and death with COVID-19: Evidence from a large population based cohort study	Meng 2023	UK Biobank adults aged 37–73 reinvited for SARS-CoV-2 antibody testing (general population; not a patient-only cohort)	Habitual glucosamine user	Baseline 2006–2010; outcomes from Jan 30, 2020; median follow-up 1.67 years	SARS-CoV-2 infection	Fully adjusted OR=0.96 (0.92–1.01), p=0.08; total events=15299	163031	42673	COVID-19 hospital admission	Fully adjusted HR=0.80 (0.74–0.87), p<0.001; total events=4214	163031	42673	COVID-19 mortality	Fully adjusted HR=0.81 (0.69–0.95), p=0.01; total events=1141	163031	42673								
4	Associations of regular glucosamine use with all-cause and	Li 2020	UK Biobank general population;	Regular glucosamine supplement use	Recruitment 2006 –	All-cause mortality	HR=0.85 (0.82–0.89);	16665	3217	CVD mortality	HR=0.82 (0.74–0.90);	3202	600	Cancer mortality	HR=0.94 (0.88–0.99);	6571	1519	Respiratory disease	HR=0.73 (0.66–0.81	2917	463	Digestive disease	HR=0.74 (0.62–0.90	914	147

	cause-specific mortality: a large prospective cohort study		baseline 2006–2010		2010; median follow-up 8.9 years		p<0.001				p<0.001				p=0.031			mortality	); p<0.001			mortality	); p<0.001		
5	Glucosamine and Chondroitin Use and Mortality Among Adults in the United States from 1999 to 2014	Bhimani 2023	NHANES 1999–2014 adults aged ≥20 (US civilian noninstitutionalized); excluded pregnancy, rheumatoid arthritis, ineligible mortality follow-up, and	Regular glucosamine use (≥20 days in past 30 days)	Baseline 1999–2014; mortality follow-up via NDI through end of 2015 (median follo	All-cause mortality (glucosamine)	HR=1.02 (0.86–1.21)	4696	204	Cancer mortality (glucosamine)	HR=1.19 (0.83–1.71)	1028	55	Cardiovascular mortality (glucosamine)	HR=0.72 (0.46–1.15)	815	24	Other-cause mortality (glucosamine)	HR=1.03 (0.83–1.28)	2840	125	All-cause mortality (chondroitin)	HR=1.04 (0.87–1.25)	4748	156

			missing supplement info		w-up 8.1 years)																				
6	Glucosamine/Chondroitin and Mortality in a US NHANES Cohort	King 2020	NHANES 1999–2010 US adults aged ≥40; excluded participants with missing vital status or baseline cardiovascular disease (CVD)	Glucosamine/chondroitin use ≥365 days before interview (user)	Baseline 1999–2010; mortality follow-up via 2015 Linked Mortality File to Dec 31, 2015	All-cause mortality	HR=0.73 (0.57–0.93); p=0.01; deaths=3366	16028	658	CVD mortality	HR=0.42 (0.23–0.75); p=0.004; deaths=674	16028	658												

					(median follow-up 107 months)																				
7	Glucosamine use and risk of colorectal cancer: results from the Cancer Prevention Study II Nutrition Cohort	Kantor 2018	CPS-II Nutrition Cohort, US adults; excluded prior CRC, inflammatory bowel disease (RA, UC, Crohn's), loss to follow-up, unverifiable cases	Current regular users (≥4 days/week), modeled as time-varying	Baseline 2001; follow-up to June 30, 2011 (mean ~8.3 years)	Baseline current use vs never	HR=0.88 (0.75–1.03)	1148	195	708,990 / 158,267	Baseline duration ≤2 yrs vs never	HR=0.82 (0.68–1.00)	1148	121	708,990 / 104,079	Baseline duration ≥3 yrs vs never	HR=0.99 (0.78–1.26)	1148	74	708,990 / 54,188	Updated current use vs never	HR=0.83 (0.71–0.97)	1034	210	617,130 / 178,254

Supplementary Table 4 NOS extension

Study (Author, Year)	Representativeness	Non-exposed selection	Exposure ascertainment	Outcome absent at baseline	Adjusted for age/sex	Adjusted for other confounders	Outcome assessment	Follow-up length	Follow-up adequacy	Total
Zhou 2022	★	★	★	★	★	★	★	★	★	9
Liu 2022	★	★	★	★	★	★	★		★	8
Meng 2023	★	★	★	★	★	★	★		★	8
Li 2020	★	★	★	★	★	★	★	★	★	9
Kantor 2022	★	★	★	★	★	★	★	★	★	9
Bhimani 2023	★	★	★	★	★	★	★	★	★	9
Kantor 2018	★	★	★	★	★	★	★	★	★	9
Zhang 2022	★	★	★	★	★	★	★	★	★	9
King 2020	★	★	★	★	★	★	★	★	★	9
Xu 2025	★	★	★	★	★	★	★		★	8
Li 2023	★	★	★	★	★	★	★	★	★	9
He 2025	★	★	★	★	★	★	★		★	8
Li 2022	★	★	★	★	★	★	★	★	★	9
Xu 2022	★	★	★	★	★	★	★	★	★	9
Bell 2012	★	★		★	★	★	★	★		7
Brasky 2011	★	★		★	★	★	★	★		7
Kantor 2013	★	★		★	★	★	★	★	★	8
Kantor 2016	★	★		★	★	★	★	★	★	8
